# Thymoquinone Suppresses Cell Proliferation and Enhances Apoptosis of HL60 Leukemia Cells through Re-Expression of *JAK/STAT* Negative Regulators

**DOI:** 10.31557/APJCP.2021.22.3.879

**Published:** 2021-03

**Authors:** Belal Almajali, Hamid Ali Nagi Al-Jamal, Wan Rohani Wan Taib, Imilia Ismail, Muhammad Farid Johan, Abd Almonem Doolaanea, Wisam Nabeel Ibrahim, Syed Ahmad Tajudin

**Affiliations:** 1 *School of Biomedicine, Faculty of Health Sciences, Universiti Sultan Zainal Abidin (UniSZA), 20300 Terengganu, Malaysia. *; 2 *Department of Haematology, School of Medical Sciences, Universiti Sains Malaysia, Kubang Kerian, Kelantan, Malaysia. *; 3 *Pharmaceutical Technology Department, Faculty of pharmacy, International Islamic University Malaysia, Kuantan, Malaysia. *; 4 *Department of Biomedical Sciences, Collage of Health sciences, QU Health, Qatar University, Doha, Qatar. *; 5 *Centralized Laboratory Management Centre, Universiti Sultan Zainal Abidin, 22200 Besut, Terengganu, Malaysia. *

**Keywords:** Thymoquinone, leukemia, JAK/STAT signaling, negative regulators

## Abstract

**Objective:**

The natural compound, thymoquinone (TQ) has demonstrated potential anticancer properties in inhibiting cell proliferation and promoting apoptosis in myeloid leukemia cells, breast cancer cells, and others. However, the effect mechanism of TQ on AML cells still not fully understood. In this study, the authors examined the effects of TQ on the expression of *JAK/STAT*-negative regulator genes* SOCS-1, SOCS-3,* and *SHP-1*, and their consequences on cell proliferation and apoptosis in HL60 leukemia cells.

**Methods:**

MTT and trypan blue exclusion tests were conducted to determine the 50% inhibitory concentration (IC50) and cell proliferation. FITC Annexin and Guava^®^ reagent were used to study the cell apoptosis and examine the cell cycle phases, respectively. The expression of *JAK/STAT-*negative regulator genes, *SOCS-1, SOCS-3*, and *SHP-1*, was investigated using reverse transcriptase- quantitative PCR (RT-qPCR).

**Results:**

TQ demonstrated a potential inhibition of HL60 cell proliferation and a significant increase in apoptotic cells in dose and time-dependent manner. TQ significantly induced cycle arrest at G0-G1 phase (P < 0.001) and enhanced the re-expression of *JAK/STAT*-negative regulator genes.

**Conclusion:**

TQ potentially inhibited HL60 cell proliferation and significantly increased apoptosis with re-expression of *JAK/STAT*-negative regulator genes suggesting that TQ could be a new therapeutic candidate for leukemia therapy.

## Introduction

Acute myeloid leukemia (AML) is a type of blood cancer characterized by the accumulation of malignant myeloid cells in the bone marrow and blood (Zhou et al., 2016). Up to date, AML patients are treated by intensive chemotherapy. However, besides the high cost of chemotherapy and its side effects, there is a low five-year survival rate in patients of 20 years and older (Sheridan 2017), and 50% of cases developed resistance (Lucas et al., 2008; Nik et al ., 2010). Therefore, alternative cheap, available, and low side effect treatment option is vitally needed.

Natural compounds are essential sources of new anticancer drugs due to their modulating effects on apoptosis and cell cycle in cancer cells with a harmless impact on healthy cells (Fulda, 2010). Thymoquinone (TQ) is one of the essential bioactive ingredients in Nigella sativa and has demonstrated potential anticancer properties (Kundu et al., 2014). Several studies have shown that TQ has inhibited cell proliferation and enhanced apoptosis in myeloid leukemia cells (El-Mahdy et al., 2005), breast cancer cells (Yıldırım et al., 2019), and multiple myeloma (Siveen et al., 2014; Li et al., 2010). However, the effect mechanism of TQ on AML cells still not fully understood. 

Janus kinase/signal transducer and activator of transcription (JAK/STAT) pathway plays a critical role in cell events and is activated by growth factors and cytokines (Raychaudhuri and Raychaudhuri 2017). Constitutive activation of JAK/STAT signaling pathway promotes the carcinogenesis and contributes to the development of resistance to tyrosine kinase inhibitors in leukemia cells (Al-Jamal et al., 2018). Therefore,* JAK/STAT *signaling pathway is a promising therapeutic target in several cancers, including leukemia (Pencik et al., 2016). *JAK/STAT *signaling is negatively regulated by tumor suppressor genes such as protein tyrosine phosphatase (SHP-1) and suppressor of cytokine signal transduction family (SOCS), particularly *SOCS-1* and *SOCS-3* (Stec et al., 2013: Tabassum et al., 2019). Additionally, *JAK/STAT* signaling pathway is constitutively activated due to epigenetic silencing of its negative regulator genes (Beldi-Ferchiou et al., 2017). 

The present study aimed to evaluate the effect mechanism of TQ on HL60 leukemia cells. For this purpose, cytotoxicity, apoptosis, cell cycle assays, and gene expression analysis of* JAK/STAT*-negative regulator genes were performed before and after treating HL60 leukemia cells with TQ.

## Materials and Methods

*Reagents*

Thymoquinone (purity ≥98%) was purchased from Sigma-Aldrich (Munich, Germany). RPMI-1640 (RPMI), 1% penicillin/streptomycin, and MTT (3-(4, 5-dimethyl thiazol-2yl)-2, 5-diphenyl tetrazolium bromide) were obtained from Nacalai Tesque (Kyoto, Japan). Fetal bovine serum was purchased from Tico Europe (Holland). Trypan blue (0.4%) was purchased from Sigma Chemicals (St. Louis, Missouri, USA). FITC Annexin V apoptosis detection kit was purchased from BD Bioscience (California, USA). Guava^®^ Cell Cycle Reagent Kit was purchased from Millipore (Germany). ReliaPrep™ RNA Cell Miniprep System and GoTaq 2-Step RT-qPCR System were purchased from Promega (USA).

*Thymoquinone preparation*

Thymoquinone stock solution was prepared in dimethyl sulfoxide (DMSO) as 5 mM, then appropriate working solutions were prepared by diluting with complete RPMI-1640 culture medium (DMSO concentration in culture media was < 0.1%). 

*Cell line and growth media*

HL60 leukemia cells were purchased from (Elabscience-Biotech Co. Ltd, Wuhan, China) and sub-cultured in T-25 culture flasks containing RPMI, supplemented with 10% of fetal bovine serum and 1% penicillin/streptomycin at a density of 5 × 10^4^ cells/mL in a humid incubator with 5 % CO_2_ at 37°C. The media is changed every 3 to 4 days to maintain cell nutrition until cells reach 70% confluence. Then the leukemia cells were sub-cultured with or without treatment according to the experiment design for different assays.

*Cytotoxicity assay*

HL60 leukemia cells were seeded in 96-well plates at a density of 2 × 10^4^ viable cells/100 μL/well, and treated with 1, 2, 3, 4, 5, and 6 µM of TQ. The plate was then incubated in a humidified incubator at 37°C with 5 % CO_2_ for 24, 48, and 72 h. MTT was dissolved to a concentration of 5 mg/ml. Twenty µl MTT solution was transferred to each well to yield a final volume of 220 µl/well. Plates were incubated for 4 h at 37˚C in 5% CO_2 _then supernatants were discarded, and 150 µl DMSO was added. Plates were then placed on an orbital shaker for 15 min, and the absorbance was recorded using TECAN Infiniti plate reader (TECAN, Männedorf, Switzerland) at 590 nm. The half maximal inhibitory concentration (IC_50_) values were calculated using GraphPad Prism 8.4.3 (San Diego, California, USA). Each experiment was performed in triplicate. 

*Viability assay*

Trypan blue dye exclusion test was used to determine the viable HL60 leukemia cells after treatment with serial concentrations of TQ. Briefly, 5×10^4^ cells/well were seeded in 6-well plates and incubated with 2, 3, and 4 µM TQ in the humidified incubator at 37°C with 5% CO_2_ for 24, 48, and 72 h. The cells were collected and centrifuged for 5 min at 500 x g, and resuspended in 200 μl phosphate-buffered saline (PBS). Then 10 µl of 0.4% trypan blue was mixed with 10 µl cell suspension for manual cells count using a hemocytometer (Hirschmann GmbH, Germany) and an inverted microscope (Olympus, Tokyo, Japan). Each procedure was repeated at least three times.

*Apoptosis assay*

FITC Annexin V apoptosis detection kit was used according to the manufacturer’s instructions and detected by flow cytometry (Beckman Coulter, Inc., CA, USA) then analyzed by CyExpert software (Beckman Coulter, Inc., CA, USA). Briefly, HL60 leukemia cells were incubated with 1, 2, and 3 µM TQ in a 6-well plate for 24, 48, and 72 h and washed with phosphate-buffered saline (PBS). Cells were then collected and resuspended in binding buffer (0.1 M Hepes/NaOH (pH 7.4), 1.4 M NaCl, 25 mM CaCl_2_), followed by incubation with FITC Annexin V and propidium iodide (PI) for 15 min in dark at room temperature prior to flow cytometric analysis. Each experiment was performed in triplicate.

*Cell cycle analysis*

Each phase of the cell cycle was evaluated using DNA flow cytometry analysis. Guava^®^ Cell Cycle Reagent Kit containing propidium iodide was used to determine the cell-cycle phase distributions. The HL60 leukemia cells were treated with 1, 2, and 3 µM of TQ for 24, 48, and 72 h, then washed with PBS and fixed with 70% ethanol for 24 h. The cells were incubated with PI dye for 30 min at 37˚C. The assay was performed using CytoFLEX Flow Cytometry (Beckman Coulter) according to the manufacturer’s instructions and analyzed by CyExpert software (Beckman Coulter). Each experiment was performed in triplicate. 

*RNA extraction*

Total RNA was extracted from TQ-treated and untreated HL60 leukemia cells after incubation for 48 h using the ReliaPrep™ RNA Cell Miniprep System following the manufacturer’s instructions. RNA purity and concentration were measured by a NanoPhotometer^®^ NP80 (Implen GmbH, München, Germany).

*Reverse transcription quantitative polymerase chain reaction (RT-qPCR)*

GoTaq 2-Step RT-qPCR System was used to synthesize cDNA from RNA samples (100 ng) according to the manufacturer’s protocol. All PCR amplifications were conducted using 2 µl of cDNA in 50 µl of GoTaq PCR master mix, following a 2-step amplification protocol: A starting denaturing step for 2 min at 95˚C, followed by 40 cycles of denaturation for 15 sec at 96˚C, and then annealing and extension for 1 min at 60˚C using a StepOne RT-qPCR Systems (Applied Biosystems). Data were analyzed by StepOne Software v2.3 (Applied Biosystems). Beta actin (β-actin) was used as a reference gene. The fold changes of gene expression levels were evaluated by relative quantification of 3 target genes, *SOCS-1*, *SOCS-3*, and *SHP-1* using the 2^-ΔΔCq^ method (Livak and Schmittgen 2001). The primer sequences are listed in Table 1. All experiments were performed in triplicates.

*Statistical analysis*

Kruskal–Wallis and Mann-Whitney tests were conducted for statistical analysis using GraphPad Prism 8.4.3 (San Diego, California, USA) and P< 0.05 was considered as significant. 

## Results

*Thymoquinone potentially inhibits HL60 cell proliferation*

**Figure 1:**
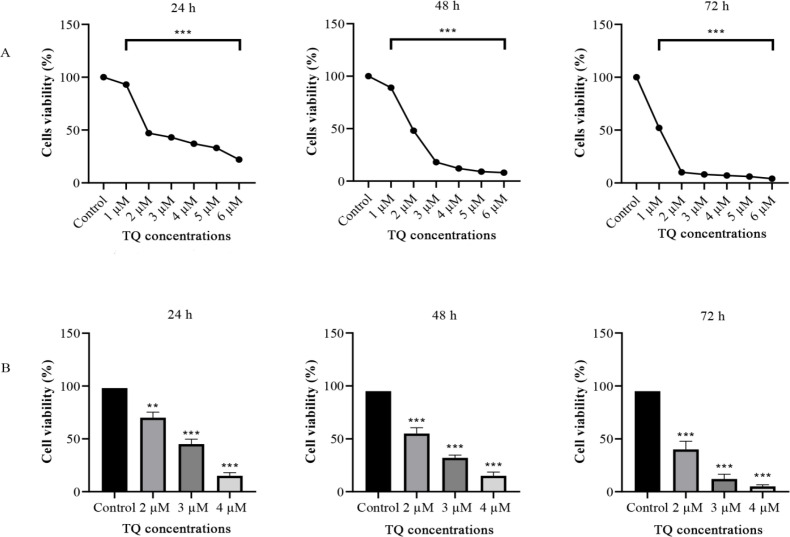
TQ Reduces the Viability of HL60 Leukemia Cells. Cytotoxicity of TQ and cell viability were assessed by MTT (A) and trypan blue staining (B) for 24, 48, and 72 h. The IC50 were 2, 2, and 1 µM, respectively. The increase in dose concentrations directly relates to the inhibition of HL60 leukemia cells. The values are expressed as mean ± SEM. Experiments were repeated at least three times. **p < 0.002 and ***p < 0.001 indicated statistical significance

To examine the cytotoxicity of TQ on HL60 leukemia cells, the cells were treated with different concentrations of TQ and incubated for 24, 48, and 72 h. The IC_50_ of TQ on HL60 leukemia cells were 2 µM after 24 and 48 h, while it was only 1 µM after incubation for 72 h. The results of MTT showed a significant inhibition of viable cells (p < 0.001) with dose and time-dependent manner (Figure 1A).

*Thymoquinone induces apoptosis and cell-cycle arrest at the G1/S Phase*

**Figure 2:**
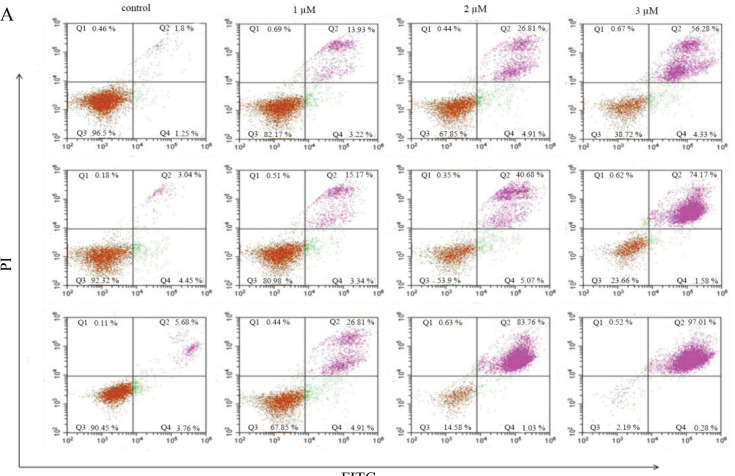
Effectiveness of TQ on HL60 Leukemia Cells Apoptosis. The apoptotic activity of HL60 leukemia cells after treatment with 1, 2, and 3 µM of TQ for 24, 48, and 72 h. Cells were stained by FITC Annexin V and PI and analyzed by flow cytometry (A). The percentage of cell death based on the estimation of apoptosis in different treatments (B). Time and dose-dependent increase in apoptotic activity was observed. Data were presented as mean ± SEM. (p < 0.001).

The vitality and fraction of apoptotic and necrotic HL60 leukemia cells were obtained by FITC annexin V and flow cytometry analysis (Figure 2). Based on the incubation of cells with 1, 2, and 3 µM TQ for 24, 48, and 72 h, there was a significant reduction of cell viability at all TQ concentrations for all incubation periods compared to that of untreated HL60 leukemia cells. The result revealed that after cells incubation with 1 µM TQ, the apoptotic cells were significantly increased (14%) compared to that in untreated cells (2%). By increasing TQ concentration, there was a dramatically increased in apoptosis to reached 56%, 74% and 97% at 3µM after incubation for 24, 48 and 72h, respectively.

**Figure 3:**
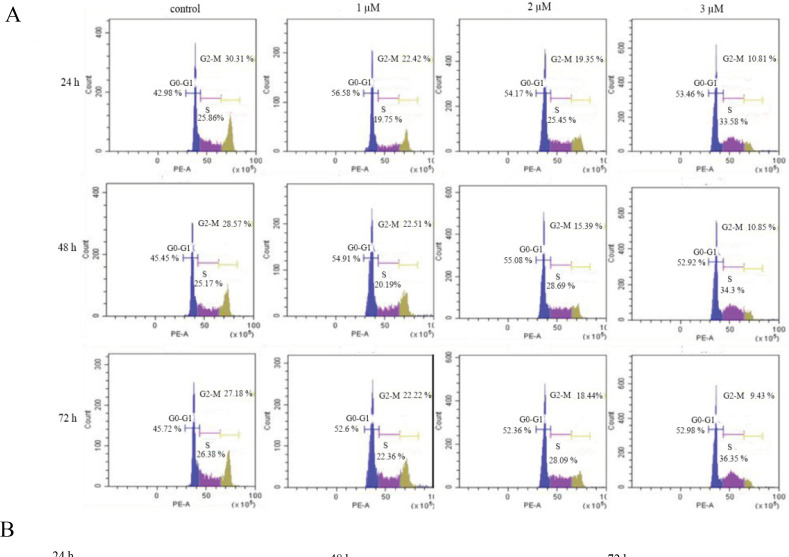
The Cell Cycle Distribution of HL60 Leukemia Cells after Treatment with TQ. Flow cytometric analysis of cell cycle changes after exposure to 1, 2, and 3 μM of TQ for 24, 48, and 72 h. The distribution of the cells was determined using flow Cytometry (A). The percentage of cells in different stages of the cell cycle with respect to control (B). Data were presented as mean ± SEM. (p < 0.001).

The effect of TQ on cell cycle progression of HL60 leukemia cells was investigated after incubation with 1, 2, and 3 µM TQ for 24, 48, and 72 h. The results showed a significant increase in G0-G1 and S phase by increasing TQ concentration with the highest percentages, 53% and 36% for G0-G1 and S phase, respectively at 3µM TQ for 72 h (Figure 3). However, the percentage of cells in G2-M phase was markedly reduced by increasing TQ doses.

*Thymoquinone enhances re-expression of JAK/STAT- negative regulators*

**Figure 4:**
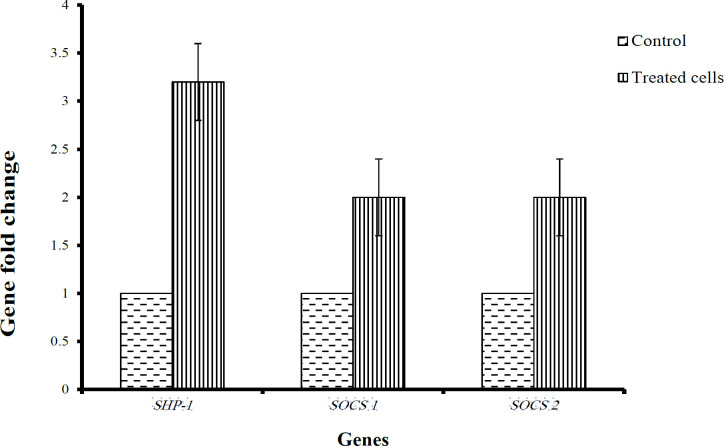
RT-qPCR of Negative Regulators of JAK/STAT Signaling in HL60 Leukemia Cells. The relative normalized ratio of RT-qPCR revealed that TQ significantly up-regulates the expression of targeted genes in treated cells. *SHP-1 *is re-expressed in treated cells more than 3 fold, while *SOCS1* and *SOCS3* are re-expressed 2-fold higher compared with control. Data were presented as mean ± SEM. (p < 0.001).

The results revealed a significant up-regulation of SOCS-1 and SOCS-3 by a two-fold change compared to untreated cells, while SHP-1 was upregulated for more than three-fold change (Figure 4). These results indicated that TQ effectively re-expressed JAK/STAT-negative regulators.

**Table 1:** Nucleotide Sequences of the Primers Used in RT-qPCR Study

Gene	Primer name	Nucleotide sequence (5´–3´)	Reference
*SOCS1*	SOCS1 F	GACGCCTGCGGATTCTAC	Hadroj et al., 2018
	SOCS1 R	AGCGGCCGGCCTGAAAG	
*SOCS3*	SOCS3 F	GACCAGCGCCACTTCTTCAC	Musalli et al., 2019
	SOCS3 R	CTGGATGCGCAGGTTCTTG	
*SHP-1*	SHP-1 F	GCCTGGACTGTGACATTGAC	Samarghandian et al., 2019
	SHP-1 R	ATGTTCCCGTACTCCGACTC	
*β-actin*	β -actin F	CTGGCACCCAGGACAATG	Relles et al., 2016
	β -actin R	GCCGATCCACACGGAGTA	

## Discussion

Up to date, the standard treatment of several cancers including AML is chemotherapy. However, the side effects of chemotherapy and its relapse represent the significant challenges in the treatment of AML patients. Therefore, searching for alternative safer, cheaper, more effective and available treatment is crucially needed. TQ has inhibited cell proliferation and enhances apoptosis in many cancers cells, including AML cells (Hodroj et al., 2018; Musalli et al., 2019). In the present study, the effect of TQ on the expression of *JAK/STAT*-negative regulator genes in *HL60* leukemia cells was evaluated. 

The IC_50_ of TQ on HL60 leukemia cells was 2 µM after 24 and 48 h of incubation with TQ but it was only 1 µM after 72 h. Our findings agreed with that previously reported in which the IC50 of TQ on A549 lung cancer cells was determined after exposure for 72 h and was significantly decreased compared to 24 h of cell exposure to TQ (Samarghandian et al., 2019). The proliferation of Mia PaCa-2 human pancreatic cancer cells was notably inhibited by increasing TQ concentration and prolonged exposure with the highest inhibition of the cell proliferation at 50 µM for 72 h of exposure compared to 24 and 48 h (Relles et al., 2016). In similarity, the proliferation assay results of the present study showed a decline in living HL60 leukemia cells after treatment with 1 µM TQ to be 93% compared to only 23 % viable cells after treatment with 6 µM TQ for 24 h. On the other hand, the percentages of viable HL60 leukemia cells were significantly decreased to 10, 8 and 7% after incubation for 72 h compared to 47, 44 and 38 %, respectively after 24 h for the same concentrations of TQ (2, 3, 4 µM) suggesting that the effect of TQ on proliferation of HL60 leukemia cells is time and dose-dependent. 

Additionally, the expression of *JAK/STAT*-negative regulator genes, *SHP-1, SOCS-1*, and *SOCS-3* genes, was investigated before and after treating HL60 leukemia cells with TQ. Interestingly, the findings of the present study showed that the inhibition of HL60 cell proliferation was associated with re-expression of *SHP-1, SOCS-1*, and *SOCS-3* genes that function as tumor suppressor genes through negative regulation of* JAK/STAT* pathway (Al-Jamal et al., 2018; Wonganan et al., 2017). Our findings are supported by previous findings in which cell proliferation inhibition of breast cancer cells was associated with upregulation of *P53* tumor suppressor gene after treatment with TQ (Dastjerdi et al., 2016). Moreover, re-expression of *SHP-1, JAK/STAT*-negative regulator gene was associated with suppression of *STAT3 *signaling and cell proliferation inhibition (Al-Jamal et al., 2015). *SOCS-1* and *SOCS-3* genes are also negative regulators of* JAK/STAT* pathway, were down-regulated in MV4-11 leukemia cells and their re-expression by demethylation was associated with inhibition of JAK/STAT signaling and lower cell proliferation (Al-Jamal et al., 2015). In similarity, our findings revealed that the cell proliferation inhibition of HL60 leukemia cells was associated with a significant re-expression of *SHP-1, SOCS-1*, and *SOCS-3* genes after exposure to 2 µM TQ. These findings suggest that TQ inhibited cell proliferation through re-expression of *JAK/STAT*-negative regulators resulting in inhibition of *JAK/STAT *signaling. 

Besides, the effect of TQ on HL60 cell apoptosis was assessed in the present study and the results showed that TQ effectively induced apoptosis with a significant increase in the apoptotic cells by increasing the dose of TQ in all exposure periods. The highest apoptosis was detected after 72 h of exposure (97%) compared to 56% and 74% apoptotic cells after 24 and 48 h respectively, with the same TQ concentration. These findings suggest that the effect of TQ on apoptosis in HL60 leukemia cells is time and dose-dependent. These results were similar to that reported by Amin Soltani and colleagues (Soltani, et al., 2017) in which TQ enhanced apoptosis in Jurkat leukemia cells with time and dose-dependent manner. 

Furthermore, TQ showed anti-proliferative activity by inducing cell-cycle arrest at the G0/G1 and S phase associated with re-expression of the tumor suppressor protein (Gali-Muhtasib et al., 2008; Al-Jamal et al., 2018). In consistency, the findings of the present study indicate that TQ induced cell cycle arrest of HL60 leukemia cells at G1 and S phases that were 46% and 27%, respectively before treatment and increased significantly to become 53% and 37% after treatment with 3 µM TQ for the same period of incubation. The results also showed that TQ induced HL60 cell cycle arrest at S phase in time and dose-dependent manner.

Taken together, the findings of the present study indicate that TQ induced cell proliferation inhibition of HL60 leukemia cells with a cell cycle arrest in early stages, and apoptosis induction associated with re-expression of *SHP-1, SOCS-1*, and *SOCS-3* genes. The findings suggest that the effect of TQ on apoptosis and cell cycle-arrest of HL60 leukemia cells could be due to the suppression of JAK/STAT signaling through enhanced re-expression of JAK/STAT-negative regulators. However, further study is needed to investigate the TQ effect on JAK/STAT signaling.

## Author Contribution Statement

Conceptualization, H.A. and B.A.; methodology, B.A., H.A.; software, B.A., H.A., S.J.; validation, H.A., A.D. and W.I.; formal analysis, B.A.; investigation, B.A., H.A.; resources, H.A.; data curation, B.A.; writing—original draft preparation, H.A. and B.A.; writing—review and editing, H.A., A.D., W.I., M.J., W.T., I.I.; visualization, H.A., B.A.; supervision, H.A.; project administration, H.A.; funding acquisition, H.A. All authors have read and agreed to the published version of the manuscript. 
